# Reliability of Measures of Lower-Body Strength and Speed in Academy Male Adolescent Soccer Players

**DOI:** 10.1519/JSC.0000000000004639

**Published:** 2023-10-16

**Authors:** Jack Ferguson, Neil V. Gibson, Matthew Weston, Robert McCunn

**Affiliations:** 1 Heart of Midlothian Football Club, Oriam, Scotland's Sport Performance Centre, Edinburgh, United Kingdom;; 2 City Architect and Transformational Design, Blacktown City Council, Sydney, Australia; and; 3 Institute for Sport, Physical Education and Health Science, Moray House School of Education and Sport, University of Edinburgh, Edinburgh, United Kingdom

**Keywords:** youth football, reliability, test sensitivity, eccentric hamstring strength, isometric adductor strength, sprint speed

## Abstract

Ferguson, J, Gibson, NV, Weston, M, and McCunn, R. Reliability of measures of lower body strength and speed in academy male adolescent soccer players. *J Strength Cond Res* 38(3): e96–e103, 2024—The Nordbord and ForceFrame represent a practical and time efficient means of assessing eccentric hamstring and isometric adductor strength in the large number of squads and players associated with youth soccer academies, yet measurement reliability in this population is unexamined. Therefore, over a period of 4 days, with no less than 24 hours and no more than 48 hours between trials, 37 players (age: 14.7 ± 0.8 years, stature: 168.7 ± 7.8 cm, mass: 57.7 ± 9.1 kg, and maturity offset: 0.8 ± 0.9 years) were assessed for eccentric hamstring strength (force, torque), isometric adductor strength (long and short lever positions), and 30-m sprint (5, 10, and 20-m splits), using the Nordbord, ForceFrame, and electronic timing gates, respectively, on 3 separate occasions. Relative reliability (intraclass correlation coefficient) was rated as good for all Nordbord (range: 0.86–0.89) and ForceFrame (0.78–0.85) measures and ranged from moderate (0.53) to excellent (0.93) for the speed measures, improving with increased distance. Absolute reliability (standard error of the measurement [%*SEM*]) ranged from 7 to 8% (Nordbord), 3 to 11% (ForceFrame), and 1 to 4% (sprints). Our data provide the first Nordbord and ForceFrame reliability estimates in adolescent soccer academy players. To interpret test sensitivity, practitioners are encouraged to interpret our estimates of absolute reliability against meaningful change values derived from personal experience and evidence-based knowledge and not against absolute or standardized thresholds.

## Introduction

In soccer, weekly training loads are periodized to minimize fatigue and maximize physiological adaptation (28). Monitoring load response is therefore an important part of the training process as it can help maximize performance, enhance fitness, reduce injury, and evaluate training plans (44). Furthermore, in adolescent soccer players, monitoring training responses and adapting training accordingly can aid talent development by informing how physical, technical, and tactical training is scheduled, particularly when transitioning from part-time to full-time training regimes (45).

The importance of speed in soccer is well established (15). In the context of youth soccer, regular monitoring of a player's sprinting can aid talent identification and long-term athlete development by informing progression-related decision-making (38) and measurement over a range of distances provides information on acceleration ability (e.g., 5–10 m) and maximal velocity (20–40 m) (10). Furthermore, linear sprint speed is sensitive to soccer-specific loading activities (e.g., scoring a goal or making a last man tackle); therefore, assessing sprint performance using split-times is commonplace in soccer (41). However, a perceived high risk of injury by players and coaches combined with scheduling during congested in-season schedules represent barriers to regular maximal speed assessments (29).

Performance-based metrics from the countermovement jump test, such as jump height and peak power, seem inappropriate for assessing the levels of neuromuscular fatigue, and other testing metrics should be considered (5). Measures lower-body peak force and peak torque are, however, reduced up to 48‐hours post-match with variability in the speed of recovery found between individual players (17). Therefore, using strength tests to assess the levels of peak force and peak torque may be a more suitable method for monitoring the effects of training and match play of elite adolescent soccer players. Furthermore, in adolescent soccer, there is a high incidence of muscular injuries during training and match play with the hamstrings and adductors being the most commonly affected muscles (36). Assessing the strength of these muscle groups on a regular basis may therefore provide practitioners with data that can be used to alter training and match load of adolescent players to reduce the risk of injury.

The Nordbord and ForceFrame are a practical, cost-effective, easy-to-use (i.e., not constrained by tester skill and experience) (35,40), and time efficient means—an important consideration when dealing with the large number of squads and players in youth academies—of assessing eccentric hamstring and isometric adductor strength, respectively (8,12). For example, the Nordic hamstring curl is commonly used in adolescent footballers' gym sessions to increase eccentric hamstring strength (3), and testing takes ∼2 minutes per player (8). However, to support effective decisions on training content and intensity, establishing the reliability of measures used to monitor responses to daily load is crucial (31). Although Nordbord and ForceFrame reliability have been previously reported in adult populations (12,35) and the Nordbord in youth footballers aged 10–16 years who were not involved in any formal strength and conditioning training (16), reliability has yet to be established in academy soccer players. The primary objective of this study was to establish the reliability of eccentric hamstring strength and isometric adductor strength tests using the Nordbord and ForceFrame, respectively, in academy-based adolescent soccer players.

## Methods

### Experimental Approach to the Problem

A repeated measures design was used to assess the reliability of lower-body muscular strength and speed in adolescent footballers. Each player completed an eccentric hamstring strength, isometric adductor strength, and 30-m linear sprint test using the Nordbord, ForceFrame, and electronic timing gates, respectively. Testing took place on 3 separate occasions over a period of 4 days, with no less than 24 hours and no more than 48 hours between trials. As the 3 trials took place over 4 days, no performance changes were expected during the data collection period. Data were collected during a competitive, in-season microcycle where the focus of training was the maintenance of technical, tactical, and physical capabilities of the players. Given the age of our subjects, we obtained parental consent and subject consent through institutionally approved informed consent documents that detailed the purposes and procedures of our investigation. Ethical approval was granted by the School of Social Sciences at Heriot-Watt University, conforming to the Declaration of Helsinki.

### Subjects

Forty-six elite adolescent footballers agreed to participate in this study; however, owing to injury, illness, and international commitments, only 37 players (age: 14.7 ± 0.8 years, stature: 168.7 ± 7.8 cm, mass: 57.7 ± 9.1 kg, and maturity offset: 0.8 ± 0.9 years) completed all 3 trials. All players competed at a professional youth level against opposition players of the same age. The players attended the same elite youth academy and undertook three 90-minute training sessions with no alterations made to their weekly training regime as part of this study. All players had a minimum of 4 years' experience of playing soccer and a minimum of 2 years' experience of strength training.

### Procedures

All testing took place in the early evening before regular squad training. In the following order, players completed the Nordbord and ForceFrame tests indoors before the 30-m linear sprint test on an outdoor synthetic pitch. Outdoor temperature and wind conditions were similar between all three 30-m trials (temperature: 10–13° C, humidity: 86–95%, and wind: 9.9–12.4 mph). Each of the 3 trials was part of the player's routine physical assessment protocols at the club. Before testing, all players completed a self-selected warm-up.

#### Anthropometry and Maturity Offset

One week before testing, stature and seated height were measured to the nearest 0.1 cm using a Seca Alpha stadiometer (Seca Alpha, Birmingham, United Kingdom). Body mass was measured to the nearest 0.1 kg using calibrated Seca Alpha scales (Seca Alpha, Birmingham, United Kingdom). Age at peak height velocity (PHV) was estimated using the Mirwald predication equation (33). Maturity offset was calculated by subtracting the players age at PHV from their chronological age as described by Mirwald et al. (33).

#### Eccentric Hamstring Strength

**Figure 1.**
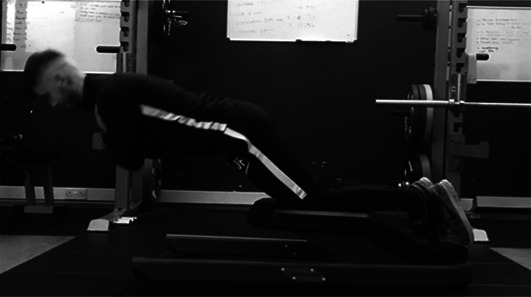
Assessment of eccentric hamstring strength using the Nordbord.

Players completed the eccentric hamstring strength test using the Nordbord (Vald Performance, Newstead, QLD, Australia), which is a padded board with 2 ankle hooks connected to force cells. Players had their ankles secured at a point superior to the lateral malleolus before completing 3 Nordic curls sustaining the contraction for as long as possible while lowering their torso toward the floor (Figure 1). Data were transmitted in real time into the researchers' smart phone using the Scorebord app (Vald Performance) before being uploaded onto the Dashbord database (Vald Performance). During each Nordic curl, data were extracted for peak force and peak torque produced by both the right and left hamstrings with the highest values imported into a Microsoft Excel spreadsheet.

#### Isometric Adductor Strength

**Figure 2.**
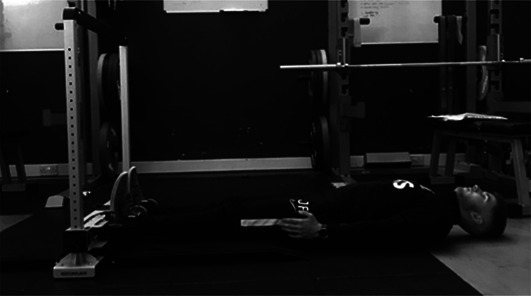
Assessment of isometric adductor strength in the long lever position using the ForceFrame.

**Figure 3.**
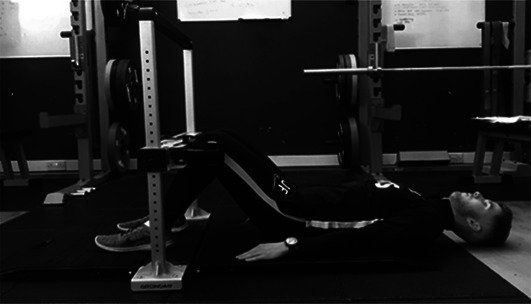
Assessment of isometric adductor strength in the short lever position using the ForceFrame.

The isometric adductor strength test was performed using the ForceFrame (Vald Performance). The ForceFrame is made up of a metal bar that can be adjusted in height dependant on the testing position desired and has 4 force pads attached to it, each connected to a force cell (34). For long lever testing, each player lay in a supine position with their knees and hips at 0°. Force transducers were positioned perpendicular to the medial malleoli, located by palpation (Figure 2). The researcher then instructed each player to complete an isometric adductor squeeze, with maximum effort, for 5 seconds. Each player completed 3 long lever efforts with approximately 10 seconds recovery. The bar was then adjusted in height for the short lever test position. Staying in a supine position, each player flexed their hips to 60° and the force transducers were set perpendicular to the medial femoral before 3, maximum effort, isometric adductor squeezes (Figure 3). Peak force for long and short lever repetitions was uploaded to the Dashbord database with the highest value, for long and short lever tests imported into a Microsoft Excel spreadsheet.

#### Sprint Performance

Distances of 5, 10, 20, and 30 m were measured and marked before the placement of electronic timing gates (Brower Timing Systems, Draper, UT) at each distance. Gates were set-up at the start of the 30-m track, and each player started the test 30 cm behind the first timing gate. Two plastic markers were placed 2 m beyond the last pair of timing gates, and each player was encouraged not to decelerate until they were past these markers. Players were allowed one practice run through the gates at ∼75% of maximum. Three maximum effort sprints were completed by each player with the fastest time for 5, 10, 20, and 30 m recorded and imported into a Microsoft Excel spreadsheet.

### Statistical Analyses

Raw data, probability density, and boxplots of Test 1, Test 2, and Test 3 for all outcome measures are visualized through Raincloud plots (1). Given our research design of more than one retest, we used to a linear model to assess for between-test changes (i.e., systematic bias) (2). Here, mixed linear models (fixed effect = test and random effect = player) were performed on all outcome measures using the *lme4* package (4) with model assumptions and performance verified using the *performance* package (27). Estimated marginal means and between-trial estimates were subsequently derived from the mixed models using the *emmeans* package (23). In the context of reliability, hypothesis testing can determine the presence of systematic bias and whether more familiarization trials are required (2), and often, the magnitude of the systematic bias is assessed by calculating standardized differences (37). While useful, a between-test comparison of means provides no indication of random variation between tests (2); therefore, we have presented between-trial differences (raw and standardized) and a visual assessment of statistical significance through the disposition of all between-trial 95% confidence intervals against the null difference (i.e., 0), but not interpreted these effects. The standard error of measurement (*SEM*), which provides an indication of the precision of a score (43) and is therefore used to understand whether the difference in measurements is real or due to error, represents our measure of absolute reliability, as per Atkinson and Nevill (2) and calculated using the *rel* package (26). Here, the *SEM* is presented not only as the overall estimate from the 3 tests and expressed primarily in raw units but also as a % through back transformation of the log-transformed between-subject *SD* (6). Relative reliability is represented by 2-way, fixed-effect model intraclass correlation coefficients (ICC_3,1_) (2,21). This model was selected as the analysis allows the researcher to apply the reliability results to a larger study with the same adolescent players (43). Analysis was performed using the *rel* package (26) with values less than 0.5, between 0.5 and 0.75, between 0.75 and 0.9, and greater than 0.90 indicative of poor, moderate, good, and excellent reliability, respectively. We acknowledge, however, that our effective sample size of 37 is lower than that needed to detect an ICC with a power of 0.80 (7), and this could influence estimate precision. Uncertainty in all our estimates is presented as 95% confidence intervals (95% CIs) and all visualizations and analyses were performed in R (version 4.1.2, R Foundation for Statistical Computing, Vienna, Austria).

## Results

### Eccentric Hamstring Strength (Force)

**Figure 4.**
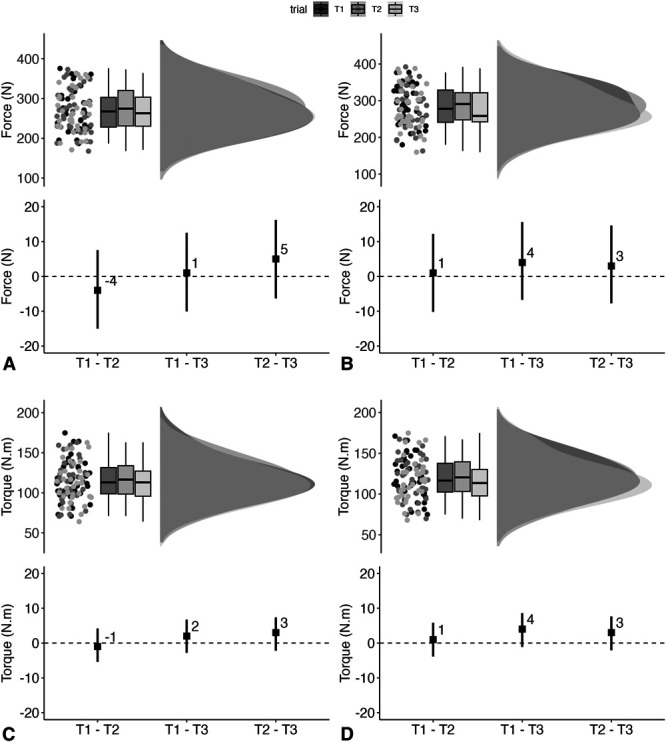
Raincloud plots, incorporating boxplots, showing Test 1 [T1], Test 2 [T2], and Test [T3] data (upper panel) along with between-trial estimates [black square] and 95% confidence intervals [black line] (lower panel) for the left leg eccentric hamstring strength force (A), right leg eccentric hamstring strength force (B), left leg eccentric hamstring strength torque (C), and right leg eccentric hamstring strength torque (D).

Left leg (Figure 4A) standardized between-trial differences ranged from −0.19 (95% CI: −0.66, 0.29) to ES 0.25 (95% CI: −0.23, 0.73). Overall *SEM* was 18 N (95% CI: 15 N, 22 N) (8%), and ICC was rated as good (0.86; 95% CI: 0.78, 0.92). For the right leg (Figure 4B), standardized between-trial differences ranged from 0.05 (95% CI: −0.42, 0.53) to 0.22 (95% CI: −0.25, 0.70). Overall *SEM* was 17 N (95% CI: 14 N, 21 N) (7%), and ICC was rated as good (0.87; 95% CI: 0.79, 0.93).

### Eccentric Hamstring Strength (Torque)

For the left leg (Figure 4C), standardized between-trial differences ranged from −0.07 (95% CI: −0.55, 0.40) to 0.31 (95% CI: −0.18, 0.79). Overall *SEM* was 8 N.m (95% CI: 7 N.m, 10 N.m) (7%), and ICC was rated as good (0.89; 95% CI: 0.81, 0.94). For the right leg (Figure 4D), standardized between-trial differences ranged from 0.11 (95% CI: −0.36, 0.59) to 0.44 (95% CI: −0.05, 0.92). Overall *SEM* was 8 N.m (95% CI: 7 N.m, 10 N.m) (7%), and ICC was rated as good (0.89; 95% CI: 0.82, 0.94).

### Isometric Adductor Strength (Short Lever)

**Figure 5.**
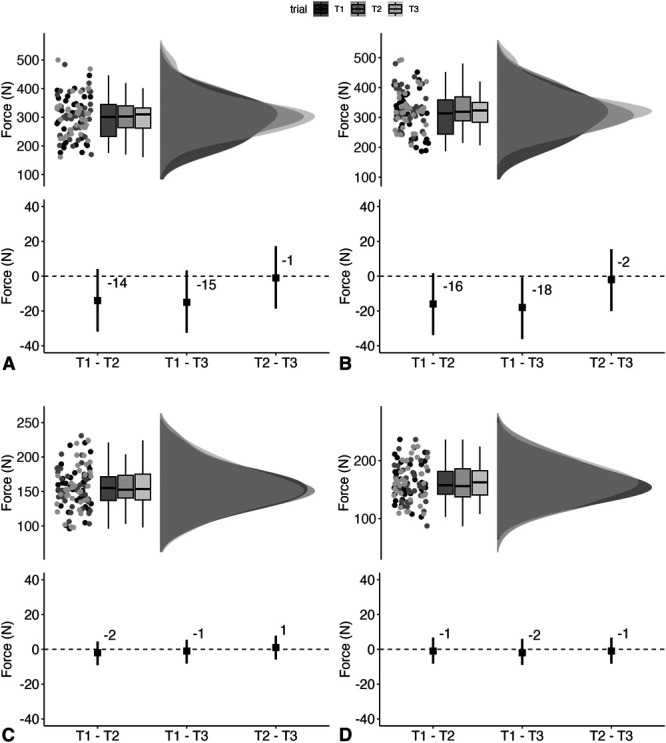
Raincloud plots, incorporating boxplots, showing Test 1 [T1], Test 2 [T2], and Test [T3] data (upper panel) along with between-trial estimates [black square] and 95% confidence intervals [black line] (lower panel) for the left adductor isometric strength short lever (A), right adductor isometric strength short lever (B), the left adductor isometric strength long lever (C), and right adductor isometric strength long lever (D).

For the left side (Figure 5A), standardized between-trial differences ranged from −0.46 (95% CI: −0.94, 0.02) to −0.02 (95% CI: −0.50, 0.45). Overall *SEM* was 30 N (95% CI: 26 N, 37 N) (11%), and ICC was rated as good (0.79; 95% CI: 0.67, 0.88). For the right side (Figure 5B) standardized between-trial differences ranged from −0.59 (95% CI: −1.10, −0.10). Overall *SEM* was 29 N (95% CI: 24 N, 36 N) (10%), and ICC was rated as good (0.78; 95% CI: 0.66, 0.87).

### Isometric Adductor Strength (Long Lever)

Left side (Figure 5C) standardized between-trial differences ranged from −0.19 (95% CI: −0.67, 0.28) to 0.08 (95% CI: −0.39, 0.56). Overall *SEM* was 12 N (95% CI: 10 N, 15 N) (8%), and ICC was rated as good (0.85; 95% CI: 0.75, 0.91). For the right side (Figure 5D), standardized between-trial differences ranged from −0.11 (95% CI: −0.59, 0.36) to −0.05 (95% CI: −0.53, 0.42). Overall *SEM* was 12 N (95% CI: 10 N, 15 N) (3%), and ICC was rated as good (0.84; 95% CI: 0.74, 0.91).

### Sprints (5–30 m)

**Figure 6.**
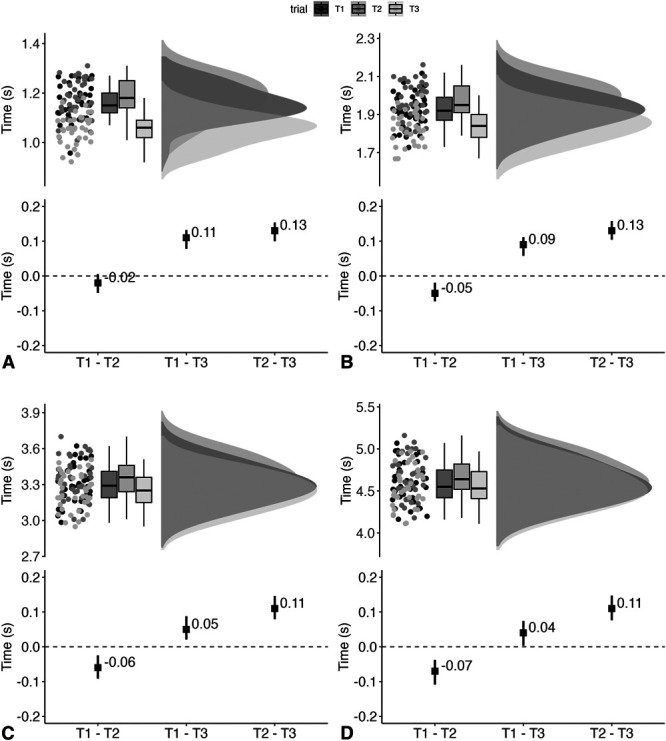
Raincloud plots, incorporating boxplots, showing Test 1 [T1], Test 2 [T2], and Test [T3] data (upper panel) along with between-trial estimates [black square] and 95% confidence intervals [black line] (lower panel) for the 5-m sprint (A), 10-m sprint (B), 20-m sprint (C), and 30-m sprint (D).

For the 5 m sprint (Figure 6A), standardized between-trial differences ranged from −0.48 (95% CI: −0.92, 0.02) to 2.63 (95% CI: 1.99, 3.27). The overall *SEM* was 0.05 seconds (95% CI 0.04 seconds, 0.06 seconds) (4%), and ICC was rated as moderate (0.53; 95% CI: 0.34, 0.70). For the 10-m sprint (Figure 6B), standardized between-trial differences ranged from −0.97 (95% CI: −1.47, −0.46) to 2.73 (95% CI: 2.02, 3.44). The overall *SEM* was 0.05 seconds (95% CI: 0.04 seconds, 0.06 seconds) (3%), and ICC was rated as moderate (0.73; 95% CI: 0.58, 0.84). Standardization between-trial differences for the 20-m sprint (Figure 6C) ranged from −0.97 (95% CI: −1.49, −0.46) to 1.88 (95% CI: 1.26, 2.50). The overall *SEM* was 0.07 seconds (95% CI: 0.06 seconds, 0.08 seconds) (2%), and ICC was rated as good (0.86; 95% CI: 0.78, 0.92). For the 30-m sprint (Figure 6D), standardized between-trial differences ranged from −1.15 (95% CI: −1.68, −0.61) to 1.75 (95% CI: 1.14, 2.37). The overall *SEM* was 0.07 seconds (95% CI: 0.06 seconds, 0.08 seconds) (1%), and ICC was rated as excellent (0.93; 95% CI: 0.89 to 0.96).

## Discussion

This study assessed the reliability of eccentric hamstring strength, isometric adductor strength, and sprint performance in academy adolescent soccer players. Although the reliability of sprint testing in academy-based soccer players has previously been reported, our study represents the first to provide reliability estimates for players when using the Nordbord and ForceFrame. Here, our adolescent eccentric hamstring strength and isometric adductor strength reliability estimates are consistent with those previously reported in adult populations.

In the context of relative reliability, all Nordbord and ForceFrame measures were rated as good, which is consistent with previous work in adults (12,35). For the sprints, relative reliability ranged from moderate to excellent and improved with increased distance. Our 10-m (0.73) and 20-m (0.86) estimates are largely consistent with those reported for youth soccer players from 11 to 17 years (range: 0.73–0.94) (13), but all sprint test estimates were worse than the those reported in adults by Shalfawi et al. (39), who found an ICC of 10, 20, and 30-m sprint performance to be 0.91, 0.91, and 0.99, respectively. Maturation status can affect running speed, with a very likely large relationship between maturation status and sprint performance reported in players who are circa-PHV (30). Therefore, fluctuations in the sprinting speed of adolescent players should be expected and may affect the reliability of associated measures.

Regarding absolute reliability, the magnitude of our *SEM* estimates for all Nordbord measures (7–8%) was largely consistent with those previously reported in young soccer players (6–8% [16]) and in adults (6–9% [35]). For the ForceFrame, our range of *SEM* estimates (3–11%) encompasses the value previously reported for adult soccer players (8% [12]). We did, however, observe higher *SEM* estimates for short lever measures. As this is the first study to quantify the reliability of isometric adductor strength from long lever positions using the ForceFrame in elite adolescent footballers, the literature provides no comparable data. However, Light and Thorborg (24) and Krause et al. (22), both observed better reliability for long lever measures of isometric adductor strength than short lever measures in adult populations. These findings may be explained by replicability of the testing position. During long lever testing, hips are kept at 0°, whereas during short lever testing, hips are flexed to 60°. The degree of hip flexion during long lever testing remained constant, within and between trials, as players were instructed to lie in a supine position with their legs straight, and any deviation from this position was rectified with verbal cues from the investigator. Such changes are harder to identify and rectify in the short lever position as the participants feet may slide forward slightly in-between efforts altering the degree of hip flexion. This may have contributed to higher *SEM* estimates. In addition, practitioners are perhaps more likely to see changes in isometric adductor strength from long lever measures due to the use of gym-based exercises that replicate this position. For example, the Copenhagen adduction exercise is commonly prescribed as it increases the strength of the adductor muscles while reducing the risk of adductor-related injuries (19). Our range of *SEM* estimates for 10-m (3%) and 20-m sprints (2%) is consistent with that reported for youth soccer players (∼2%) with the observed trend for improved absolute reliability with increased distance confirming a previous observation in elite youth Rugby Union and League players (10).

Recent work in our discipline has presented benchmarks of absolute reliability when interpreting test reliability. For example, absolute reliability was categorized “good” if the CV < 5% (25) or “acceptable” with a CV ≤ 10% (14,25). In this context, the reliability of tests in this study would be good for the sprints, acceptable for eccentric hamstring strength and adductor strength long lever but not short lever. Thresholds for absolute reliability do not represent good practice as they can lead to practitioners potentially excluding sensitive measures (32), and even the most reliable test is not necessarily a sensitive test for tracking performance changes (20). This is because small, meaningful changes (i.e., the signal) may not be distinguishable from measurement error (i.e., noise) (11), and for confidence in determining whether a change is meaningful, it is important that the signal > noise (32), that is, the change value, can be distinguished from measurement error. For example, a measurement tool may show good absolute reliability of 3%, yet if the smallest meaningful change is only 1.5%, then the test would struggle to differentiate the signal from the noise. As such, the magnitude of the measurement error needs to be interpreted in the context of usual changes and minimally important changes (9).

With this in mind, to determine measurement sensitivity, previous work interpreted the *SEM* (or typical error (SD_change_/sqrt(2)) against standardized thresholds. For example, the sensitivity of a linear sprint test in adolescent Rugby Union and League players was deemed good, OK, or marginal if the *SEM* (expressed here as the typical error) was lower, similar to, or greater than the smallest worthwhile change which was calculated as 0.2 × between-subject *SD* (10). Despite its widespread popularity, researchers and practitioners are discouraged from this approach, however, as standardization produces unrealistic values when compared values deemed realistic by expert practitioners (11). Further disadvantages of standardization are that they can be challenging to interpret and misleading depending on whether the population is homogenous or heterogeneous (18), and there is no solid basis to use a certain value for a minimally important change in standardized units as this process produces highly variable estimates (42).

Our study is not without limitation. First, the use of a self-selected warm-up does not enable replication of this activity. Second, as soccer training and match play place substantial and varied load demands on players, the use of other monitoring tests, such as change of direction, may help provide additional information on fatigue above that of speed and strength tests.

This study quantified the reliability of Nordbord, ForceFrame, and sprint performance tests in academy adolescent footballers. Our data provide the first reliability estimates in soccer academy players for eccentric hamstring strength and isometric adductor strength, as measured by the Nordbord and ForceFrame, respectively, with these values largely consistent with those reported previously in adult populations. Although force and torque showed consistent absolute reliability on the Nordbord, variations in reliability for the ForceFrame and sprint measures showed reliability to be more precise when measure using long levers and over longer distances, respectively.

**Practical Applications**

An in-house reliability study enables practitioners to quantify the amount of measurement error present in their devices. Once established, to evidence test sensitivity, measurement error should be interpreted against meaningful change and not against absolute reliability or standardized thresholds. The selection of meaningful change is challenging, will vary between practitioners, and most likely should represent a balance of practical experience and evidence-based knowledge. When using evidence-based knowledge for informing on meaningful change to interpret test sensitivity, practitioners are encouraged to seek out studies where physical change is linked to performance or injury outcomes, expert opinion on what constitutes worthwhile change, and meta-analyzed intervention effects.
